# Effect of Bridgehead Methyl Substituents on the Gas Permeability of Tröger’s-Base Derived Polymers of Intrinsic Microporosity

**DOI:** 10.3390/membranes10040062

**Published:** 2020-04-03

**Authors:** Richard Malpass-Evans, Ian Rose, Alessio Fuoco, Paola Bernardo, Gabriele Clarizia, Neil B. McKeown, Johannes C. Jansen, Mariolino Carta

**Affiliations:** 1 EaStCHEM, School of Chemistry, University of Edinburgh, Joseph Black Building, David Brewster Road, Edinburgh, Scotland EH9 3FJ, UK; R.Malpass-Evans@ed.ac.uk (R.M.-E.); ianjamesrose@gmail.com (I.R.); 2 Institute on Membrane Technology, CNR-ITM, Via P. Bucci 17/C, 87036 Rende (CS), Italy; a.fuoco@itm.cnr.it (A.F.); p.bernardo@itm.cnr.it (P.B.); g.clarizia@itm.cnr.it (G.C.); 3 Department of Chemistry, College of Science, Swansea University, Grove Building, Singleton Park, Swansea SA2 8PP, UK

**Keywords:** polymers of intrinsic microporosity (PIMs), Tröger’s base, gas separation, membrane

## Abstract

A detailed comparison of the gas permeability of four Polymers of Intrinsic Microporosity containing Tröger’s base (TB-PIMs) is reported. In particular, we present the results of a systematic study of the differences between four related polymers, highlighting the importance of the role of methyl groups positioned at the bridgehead of ethanoanthracene (EA) and triptycene (Trip) components. The PIMs show BET surface areas between 845–1028 m^2^ g^−1^ and complete solubility in chloroform, which allowed for the casting of robust films that provided excellent permselectivities for O_2_/N_2_, CO_2_/N_2_, CO_2_/CH_4_ and H_2_/CH_4_ gas pairs so that some data surpass the 2008 Robeson upper bounds. Their interesting gas transport properties were mostly ascribed to a combination of high permeability and very strong size-selectivity of the polymers. Time lag measurements and determination of the gas diffusion coefficient of all polymers revealed that physical ageing strongly increased the size-selectivity, making them suitable for the preparation of thin film composite membranes.

## 1. Introduction

Polymeric gas separation membranes are becoming an increasingly important commercial technology for gas purification as an alternative to more energy-intensive processes [1,2,3,4]. At present, membranes are utilised in hydrocarbon processing, hydrogen recovery in the manufacture of ammonia, and the separation of nitrogen from air [5,6,7,8,9]. Gas separation membranes also have potential future applications in hydrogen manufacture [10,11,12] and pre/post-combustion CO_2_ capture [13,14,15,16,17,18], and have proven to be successful for biogas upgrading [19]. A drawback of most polymeric membrane materials is their inverse relationship between selectivity and permeability. This well-known trade-off relationship has limited the use of highly selective commercial materials such as polyimides and polysulfones to small-scale applications, since their low productivity makes the process not economically feasible [20]. To compete with existing technology in large-scale applications, gas separation membranes are required to display both high permeability (*P_x_*) and selectivity (*α_xy_ = P_x_/P_y_*) for a given gas (*x*) over another gas (*y*). Robeson demonstrated that there was an upper limit for this trade-off relation in 1991 [21], based on transport parameters collected from a large number of polymers for technologically important gas pairs, proposing a semi-empirical ‘*upper-bound*’ onto plots of log *P_x_* versus log *α_xy_*, which was later confirmed in theoretical studies by Freeman [22]. The advancements in membrane technology convinced Robeson to revisit his studies in 2008 [23] when upper bounds were revised to accommodate new high performing polymers. Upper bounds were further updated for several gas pairs by Pinnau et al. in 2015 [24], and by Comesaña-Gándara et al. in 2019 [25]. The revisions were prompted by the development of Polymers of Intrinsic Microporosity (PIMs) [26,27,28,29]. PIMs are composed of highly rigid and contorted monomeric segments, which prevent the efficient packing of polymer chains in the solid state. The large fractional free-volume formed between polymer chains allows access to penetrant gases and high solubility for condensable gases such as CO_2_. At the same time, the rigid structure with impeded chain mobility enhances the energetic selectivity, resulting in a strong size-sieving character of these polymers [30]. When solvent-cast into films for gas permeability experiments, PIMs were found to combine both high selectivity with high permeabilities providing data that was used to define Robeson’s 2008 upper bounds and those proposed in 2015 and 2019. 

In 2013, we reported a novel polymerisation method to produce solution-processable PIMs from a range of aromatic diamines using Tröger’s Base (TB) chemistry [31,32,33,34,35]. In particular, two polymers—PIM-EA(Me_2_)-TB and PIM-Trip(H_2_)-TB—showed exceptional gas separation characteristics at low pressures, with a performance far above the 2008 upper bounds for several gas pairs. This was especially true for gas pairs for which the size-selectivity of the membrane is more important than solubility selectivity, such as O_2_/N_2_ or H_2_/N_2_, owing to the highly rigid structures of these polymers [29,35]. While the monomeric units from both polymers have similar bicyclic structures, the reported gas separation characteristics were significantly different. In a previous paper [29], we speculated that the bridge-head methyl groups on the ethanoanthracene unit had an influence on the gas separation process, as molecular axial rotation facilitates transport of larger gas molecules through the otherwise inaccessible free volume elements. Our suggestion was that the intermolecular interaction of the methyl groups of different polymer chains helps increase inter-chain spacing. This leads to a higher BET surface area for PIM-EA(Me_2_)-TB compared to the triptycene-based polymer but, at the same time, it also shows that the presence of the bulkier methyl groups somewhat hampers the transport of gas molecules. This behaviour suggests that PIM-Trip(H_2_)-TB [36] retains a higher proportion of smaller free volume elements, which is consistent with its enhanced size-sieving properties. The trend is clearly displayed by the relative position of PIM-EA(Me_2_)-TB in the Robeson plots, compared to the PIM-Trip(H_2_)-TB [29]. While the selectivity of PIM-Trip(H_2_)-TB drops with pressure for condensable gas pair CO_2_/CH_4_ [37], a property that may be common for similar TB polymers, the selectivity of less condensable gas pairs such as O_2_/N_2_ and H_2_/N_2_ is expected to maintain its high value over a much wider pressure range.

In this paper, we aim to fully evaluate the effects of the bridgehead methyl substituents on surface area and gas separation properties, in both ethanoanthracene (EA)- and triptycene (Trip)-based TB polymers of intrinsic microporosity (TB-PIMs).

## 2. Materials and Methods 

### 2.1. General Methods and Equipment

Commercially available reagents were used without further purification. All reactions using air/moisture sensitive reagents were performed in oven-dried or flame-dried apparatus, under a nitrogen atmosphere. Low-temperature N_2_ adsorption at 77 K and CO_2_ adsorption at 273 K, measured using a Quadrasorb Evo, (Quantachrome, Edinburgh, UK), were used to assess intrinsic microporosity (BET) and pore size distribution (PSD), respectively. The polymers were degassed at 120 °C under high vacuum overnight prior to analysis. GPC was carried out using a Viscotek GPC Max1000 system equipped with a refractive index detector and two KF-805L Shodex columns at a flow of 1 mL min^−1^ of a dilute solution of the polymer in chloroform. The TGA was performed using the device Thermal Analysis SDT Q600 (TA Instruments, West Sussex, UK)) at a heating rate of 10 °C/min from room temperature to 1000 °C. ^1^H NMR spectra were recorded in the solvent stated using an Avance Bruker DPX 400 (400 MHz,) or DPX 500 (500 MHz) instruments, Bruker, UK, with ^13^C NMR spectra recorded at 100 MHz or 125 MHz, respectively.

PIM-EA(Me_2_)-TB, [35] PIM-EA(H_2_)-TB, [36] and PIM-Trip(H_2_)-TB [29] were prepared according to the reported procedures. PIM-Trip(Me_2_)-TB was synthetized for the first time for this study (yield: 83%), according to the procedure reported in the supporting information.

Films were prepared by dissolving the PIM (0.350 g) in chloroform (20 mL) and allowing solvent to evaporate slowly over 96 h in a Teflon dish. Prior to the permeability measurements, the films were soaked in methanol for 24 h to remove residual solvent, and then dried for 24 h in air.

### 2.2. Gas Permeation Measurements

Gas permeabilities of the TB-PIMs membranes were measured in a constant volume/varying pressure apparatus at 25°. The exposed membrane area in the permeation tests was of 2.14 cm^2^ and the samples were carefully evacuated and degassed completely before the measurements with the following gases: He, H_2_, O_2_, N_2_, CH_4_ and CO_2_ (purity of 99.99+%, SAPIO, Italy).

The time lag method [38] was applied to the recorded data to determine the gas diffusion coefficient. The permeability coefficient, *P,* is reported in units of Barrer (1 Barrer = 10^−10^ cm^3^(STP) cm cm^−2^ s^−1^ cmHg^−1^) and calculated from the following equation, describing the increase in the permeate pressure, *p_t_*, in pseudo-steady state as a function to time, *t*:

(1) $$p_{t}=p_{0}+{\left(dp/dt\right)}_{0}\cdot t+\frac{RT\cdot A}{V_{P}\cdot V_{m}}\cdot \frac{p_{f}\cdot P}{l}\left(t-\frac{l^{2}}{6D}\right)$$

where *R* is the universal gas constant, *T* the absolute temperature, *A* the active area, *V_P_* the permeate volume, *V_m_* the molar gas volume at STP conditions, *p_f_* the feed pressure, *l* the membrane thickness, The initial pressure *p_0_* and the leak flow rate (*dp/dt*)_0_ are normally negligible. The last term in Equation (1) corrects for the so-called permeation time lag, Θ, which is inversely proportional to the diffusion coefficient, *D*, of the gas:

(2) $$\Theta \;=\;\frac{l^{2}}{6\;D}$$

The gas solubility coefficient, *S*, was obtained indirectly as the ratio of the permeability to the diffusion coefficient by assuming the solution-diffusion transport mechanism:

*S* = *P*/*D* (3)

Details on the used instrument, designed by Helmholz Zentrum Geesthacht and constructed by EESR (Geesthacht, Germany), can be found elsewhere [39].

## 3. Results and Discussion

### 3.1. Polymer Synthesis and Characterisation

**Figure 1:**
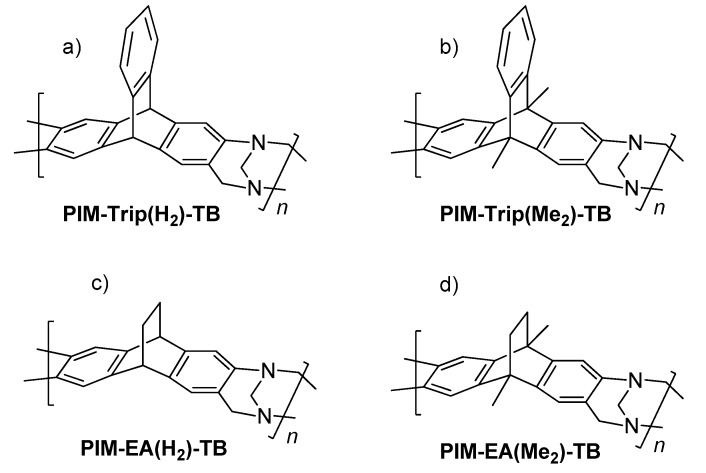
Chemical structure of the TB-PIMs: (**a**) PIM-Trip(H_2_)-TB [29], (**b**) PIM-Trip(Me_2_)-TB [This work], (**c**) PIM-EA(H_2_)-TB [36,40] and (**d**) PIM-EA(Me_2_)-TB [35,41].

To fully understand the differences in permselectivity arising from the structures of PIM-EA(Me_2_)-TB [35] and PIM-Trip(H_2_)-TB, [29] a comprehensive evaluation of the effect of methyl bridgehead substituents was planned. This was provided by preparing TB polymers from ethanoanthracene monomers, with and without methyl groups on the bridgehead (i.e., PIM-EA(Me_2_)-TB and PIM-EA(H_2_)-TB, respectively, Figure 1c,d). Similarly, polymers derived from triptycene monomers with and without bridgehead methyl substituents were prepared (i.e., PIM-Trip(Me_2_)-TB and PIM-Trip(H_2_)-TB, Figure 1a,b). 

**Scheme 1:**
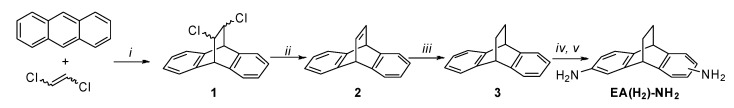
Synthesis of EA(H_2_)-NH_2_. *Reagents and conditions: i*) MW, 215 °C; 5 h; *ii*) Na, IPA/THF, reflux; *iii*) Hydrazine, Raney Ni, reflux, 24 h; *iv*) KNO_3_, TFAA, CH_3_CN, 24 h; *v*) Hydrazine, Raney-Ni^®^, THF, reflux, 16 h.

The ethanoanthracene EA(H_2_)-NH_2_ monomer was prepared following a modified procedure of Cristol et al. [42], starting from the preparation of the *cis*(*trans*)dichloro-9,10-ethanoanthracene (Scheme 1). A Diels–Alder reaction was conducted with a cheap commercial mixture of *cis/trans* 1,2-dichloroethylene and anthracene using microwave irradiation. This was followed by dehalogenation with sodium metal and hydrogenation of the obtained alkene bridge, using the catalytic decomposition of hydrazine monohydrate over Raney-Ni^®^. The hydrocarbon was nitrated with potassium nitrate/TFAA and reduced with hydrazine and Raney-Ni^®^ to obtain the EA(H_2_)-NH_2_. 

**Scheme 2:**
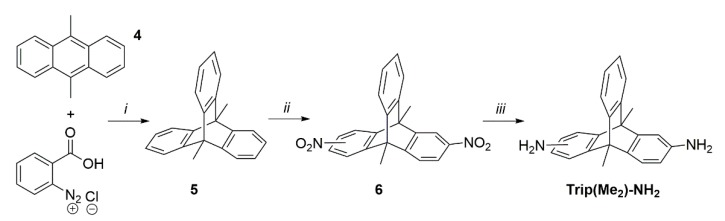
Synthesis of 2,6(7)-diamino-9,10-dimethyltriptycene Trip(Me_2_)-NH_2_. *Reagents and conditions*: *i*) DCE, 85 °C, 4 h; *ii)* KNO_3_, TFAA, DCM, CH_3_CN, 50 °C, 48 h; *iii)*. hydrazine monohydrate, Raney-Ni^®^, THF, 60 °C, N_2_, 16 h.

The synthesis of 9,10-dimethyl-diamino-triptycene [43] Trip(Me_2_)-NH_2_ monomer was performed as reported in Scheme 2, starting from 9,10-dimethylanthracene (4) and the anthranilic acid, from which we created the diazonium salt that acted as a benzyne precursor. In the same way as EA(H_2_)-NH_2_, this was followed by nitration and subsequent reduction, to obtain Trip(Me_2_)-NH_2_. 

Trip(Me_2_)-NH_2_ and EA(H_2_)-NH_2_ were reacted with trifluoroacetic acid (TFA) and dimethoxymethane (DMM), under typical TB polymerisation conditions, to form the polymers PIM-Trip(Me_2_)-TB and PIM-EA(H_2_)-TB in high yield and with high molecular mass [32]. The physical characterisation for all the reported TB-PIMs is summarised in Table 1.

The measurement of the apparent BET surface area reveals that the absence of methyl substituents on the bridgehead for PIM-EA(H_2_)-TB lead to a less microporous material than the related “methyl-containing” polymer PIM-EA(Me_2_)-TB [35]. This is most likely due to the better packing of the polymeric chains in the solid state. This confirms the hypothesis made in our 2014 study [29], where we attributed the slightly reduced surface area to the lack of methyl substituents that act as “spacer” between polymeric chains. However, in the case of PIM-Trip(Me_2_)-TB the physical characterisation displayed only a marginally higher BET surface area than its related “methyl-lacking” polymer PIM-Trip(H_2_)-TB [29].

**Figure 2:**
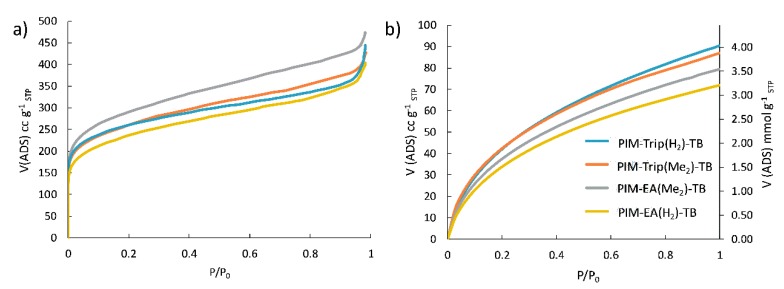
(**a**) N_2_ adsorption isotherms measured at 77 K (desorption curves are not shown for clarity); (**b**) CO_2_ uptake measured at 273 K.

For all the reported polymers, the CO_2_ sorption measurements at 273 K (Table 1 and Figure 2b) demonstrated a high total CO_2_ uptake of the TB-PIMs, while the relatively steep initial slope of the sorption curve indicates a high affinity compared to other PIMs. Indeed, gravimetric sorption measurements at 298 K have shown that PIM-EA(H_2_)-TB and PIM-Trip(H_2_)-TB have the highest CO_2_ affinity for any PIM or high free volume polymer, with the exception of Amine-PIM-1 [44]. We attribute this to the basic tertiary amines of the TB core and the ultramicroporous structures generated by the polymers. Looking at the results in detail, the CO_2_ adsorption proved higher for the triptycene-based polymers, with similar or lower total pore volume than the ethanoanthracene-based polymers. This suggests that the triptycene-based polymers undergo stronger dilation upon absorption of CO_2_, explaining the decrease in selectivity with increasing pressure as reported by Genduso et al. [37]. This is also the reason why the presence of ultramicropores leads to a favourable combination of permeability and excellent selectivity for gases with low affinity and large difference in size, such as O_2_/N_2_ and H_2_/N_2_ but not necessarily for gas pairs involving CO_2_, which may remain ‘trapped’ if the affinity for the polymer chain segments surrounding the pore are too high (see discussion below).

**Figure 3:**
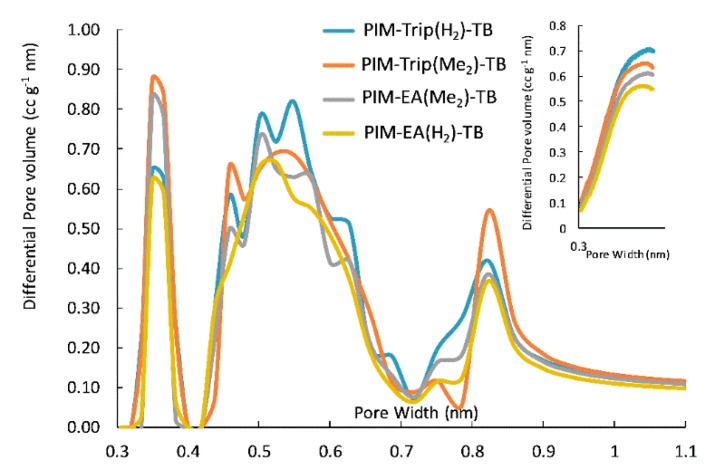
NLDFT and H-K (inset) model PSD measured from CO_2_ at 273 K.

A qualitative evaluation of the pore size distribution (PSD), obtained by CO_2_ adsorption measurement at 273 K and evaluated with NLDFT and Horvath–Kawazoe (H-K) models (Figure 3 and inset), showed the typical bimodal distribution of PIMs, with a set of very narrow pores centred at ~ 0.4 nm and a second set of slightly larger pores centred at ~ 0.6 nm. The NLDFT analysis shows a broader distribution of the ultramicropores in both Trip-based polymers than in both EA-based polymers, with the peak height in the differential pore volume changing in the order PIM-Trip(Me_2_)-TB > PIM-Trip(H_2_)-TB, and PIM-EA(Me_2_)-TB > PIM-EA(H_2_)-TB. Instead, the H-K model shows a slightly higher total ultramicropore volume in PIM-Trip(H_2_)-TB than in PIM-Trip(Me_2_)-TB, so quantitative interpretation of these results should be conducted with caution. 

### 3.2. Membrane Preparation and Gas Permeability Measurements

**Figure 4:**
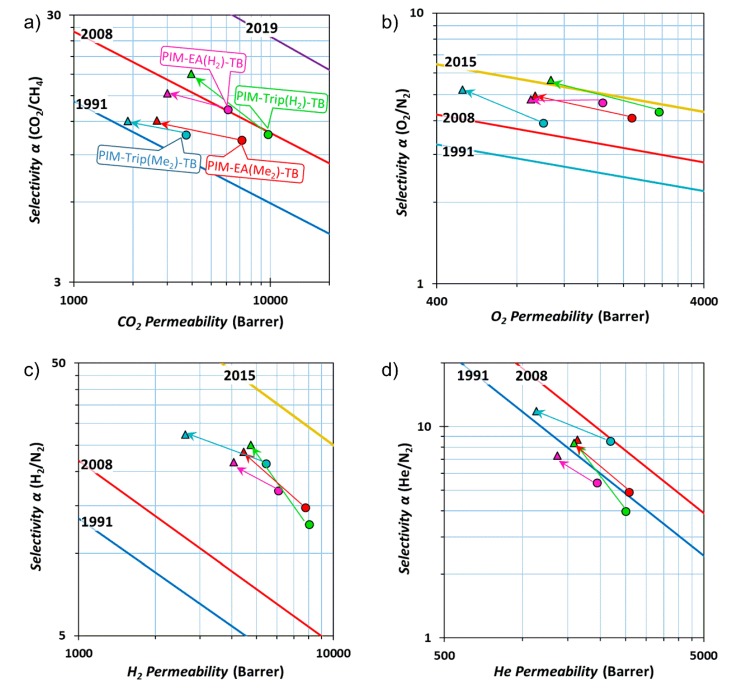
Robeson’s plots of the four TB-PIMs. Measurements at 25 °C and 1 bar feed pressure. Data are reported for methanol treated (circles) and aged (triangles) samples. Arrows indicates the change in permselectivities for aged samples of PIM-Trip(H_2_)-TB (green, 102 days), PIM-Trip(Me_2_)-TB (blue, 407 days), PIM-EA(H_2_)-TB (violet, 119 days) and PIM-EA(Me_2_)-TB (red, 106 days). Continuous lines represent the different upper bounds: 1991 [21], 2008 [23], 2015 [24], and 2019 [25].

The polymers all showed complete solubility in chloroform, allowing the preparation of robust self-standing films suitable for gas permeation measurements. Despite their higher BET surface areas, PIM-EA(Me_2_)-TB and PIM-Trip(Me_2_)-TB showed similar or lower gas permeability than their “methyl-less” counterparts (Table 2 and Figure 4). For CO_2_, the permeability of PIM-Trip(H_2_)-TB is around three times that of PIM-Trip(Me_2_)-TB. This is consistent with our previous suggestion that PIMs without methyl substituents on the bridgehead retain a higher amount of accessible free volume [29]. In contrast, PIM-EA(Me_2_)-TB has greater permeability than PIM-EA(H_2_)-TB.

In addition, the reported polymers present different permeation orders for the investigated gases, depending on the bridgehead substituents. PIM-Trip(Me_2_)-TB and, at a lower extent, PIM-EA(Me_2_)-TB, show a larger permeability for H_2_ than CO_2_. Instead, the “methyl-less” TB-PIMs display an ‘inverse selectivity’ (i.e., CO_2_ > H_2_). This is particularly evident for the PIM-Trip(H_2_)-TB, consistent with the CO_2_ uptake data at 273 K (Table 1). Only for H_2_ does PIM-EA(Me_2_)-TB prove slightly more permeable than PIM-EA(H_2_)-TB (Figure 4 and Table 2), but still far from the values of PIM-Trip(H_2_)-TB. This resulted in overall better performance for the two H_2_-bridgehead TB-PIMs for important gas pairs, surpassing the 2008 upper bound and approaching the 2015 upper bound for O_2_/N_2_, and H_2_/N_2_. As shown in Figure 4, both PIM-Trip(H_2_)-TB and PIM-EA(H_2_)-TB perform better for the important O_2_/N_2_ gas pair than the related Me-containing polymers, implying better molecular sieving, since their separation relies predominantly on diffusivity selectivity. 

Rapid physical ageing is a characteristic feature of PIMs and other superglassy polymers, which leads to a decrease in permeability, coupled with a commensurate increase of selectivity, often following the typical Robeson trade-off. In the case of several TB-PIMs, we reported previously that the increase of selectivity is often greater than the related decrease of permeability, which places their data in a more favourable area of the Robeson plots [29,33,34]. This trend is also confirmed for the PIMs in the current study and is especially evident when evaluating the data of the H_2_-bridgehead TB-PIMs. In fact, aged samples of PIM-EA(H_2_)-TB and PIM-Trip(H_2_)-TB show, once more, better performance than the related Me-bridgehead samples. In particular, despite the expected loss of permeability, physical ageing helps PIM-Trip(H_2_)-TB to surpass the 2008 upper bound for CO_2_/CH_4_. A similar trend is also observed for the H_2_/CH_4_ gas pair where, again, ageing improves the overall performance of the H_2_-bridgehead polymers, whereas PIM-EA(Me_2_)-TB and PIM-Trip(Me_2_)-TB show the opposite behaviour.

**Figure 5:**
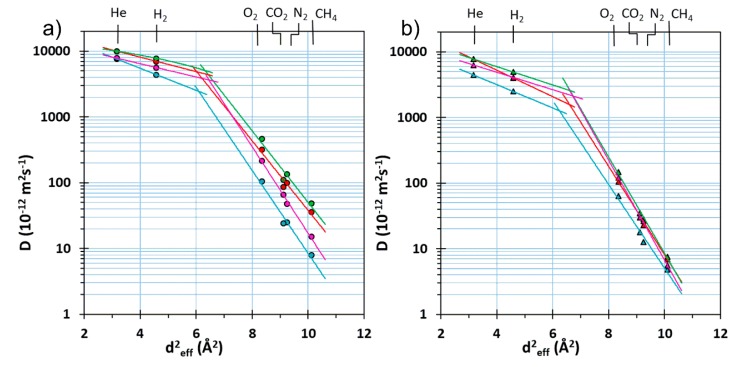
Correlation of the diffusion coefficient and effective gas diameter [46] for the MeOH-treated PIM-Trip(H_2_)-TB (green), PIM-Trip(Me_2_)-TB (blue), PIM-EA(H_2_)-TB (violet) and PIM-EA(Me_2_)-TB (red) membranes (**a**), and the same samples after ageing for 102, 407, 119 and 106 days, respectively (**b**). Lines represent the best fit of the data for helium and hydrogen, and for oxygen, carbon dioxide, nitrogen and methane, respectively. Measurements at 25 °C and 1 bar feed pressure.

For the freshly MeOH treated membranes, the diffusion coefficients for most gases decrease in the order PIM-Trip(H_2_)-TB > PIM-EA(Me_2_)-TB > PIM-EA(H_2_)-TB > PIM-Trip(Me_2_)-TB (Figure 5a). The size selectivity, expressed by the slope of the best fit for the data of oxygen, carbon dioxide, nitrogen and methane [45], increases in the same order. With exception of CO_2_ and N_2_, which are inverted in all fresh samples but PIM-EA(H_2_)-TB, the diffusion coefficient decreases with increasing effective molecular diameter as defined by Teplyakov and Meares [46]. After ageing, the diffusion coefficient decreases with the gas diameter more markedly **(**Figure 5b), evidencing a stronger size selectivity of the polymers [30]. Interestingly, PIM-Trip(H_2_)-TB maintains a relatively high diffusion coefficient for smaller gas molecules upon ageing, but it becomes the most size selective, whereas PIM-Trip(Me_2_)-TB shows exactly the opposite trend, with the lowest diffusion coefficient and the lowest size selectivity.

To summarize, the present manuscript confirms the possibility to tailor the transport properties of PIMs by the targeted use of bulky or contorted groups in the backbone, but it also shows that one of their difficulties is that even their basic properties, like the BET surface area or the total pore volume, depend on a multitude of factors and must always be used in combination with other properties to fully understand the correlation with the transport properties. In our case, all polymers have fundamentally the same backbone, and the lateral methyl groups may serve as spacers that create free volume, but if space is created by other groups, they may also fill that space. On the other hand, the triptycene groups are also expected to serve as spacers between the chains, but if they occasionally meet in an antiparallel alignment, their π-π stacking reduces the surface area with respect to individual triptycene groups. Therefore, high BET surface area and high pore volume are correlated but not necessarily in a straightforward way, because the BET area depends strongly on the pore (or better: free volume element) size distribution and interconnectivity rather than just on the total pore volume. In turn, permeability depends on both the total free volume and the free volume element size distribution, while selectivity depends more on the bottlenecks between the free volume elements [45].

## 4. Conclusions

We provide a systematic evaluation of the effect on the physical properties of methyl bridgehead substituents on the ethanoanthracene or triptycene unit for a series of TB-PIMs. Comparison of the “methyl-less” PIM-EA(H_2_)-TB and PIM-Trip(H_2_)-TB with their methyl-substituted equivalents suggests reduced overall porosity but enhanced interconnection between pores, making accessible more internal free volume. This was evaluated by both surface area and gas permeability measurements. In fact, despite the higher apparent BET surface area of PIM-Trip(Me_2_)-TB and PIM-EA(Me_2_)-TB, the overall permselectivity performance of the H_2_-bridgehead TB-PIMs proved superior for all measured gas pairs, with better molecular sieving. Ageing increases the size selectivity of the membranes, and this effect is strongest for PIM-Trip(H_2_)-TB and weakest for PIM-Trip(Me_2_)-TB. 

In conclusion, the present work shows how complex the correlations can be between physical parameters such as the total pore volume and the BET surface area, on the one hand, and gas permeability and the selectivity on the other hand. All are dependent on the total free volume or, more correctly, amount of free volume elements (FVE), but gas permeability and selectivity are also strongly dependent on pore-size distribution and pore interconnectivity (i.e., the diameter of bottlenecks between the FVEs). These, in turn, depend on the type, orientation and final packing of the bulky groups in the polymer backbone, dilation phenomena, etc. This suggests the future use of molecular simulations of three-dimensional polymer chain packing and X-ray diffraction measurements in order to further increase our understanding of the properties of these PIMs.

## Figures and Tables

**Table 1:** Summary of the physical properties of TB-PIMs polymers.

Polymer	BET Surface Area (N_2_ at 77 K) (m^2^g^−1^)	Total Pore Volume at (P/P_0_) = 0.9814 (cm^3^ g^−1^)	Mw × 10^3^ (g mol^−1^)	PDI	CO_2_ Uptake (273 K, 1 bar) cc g^−1^ (mmol g^−1^)	Ref.
PIM-EA(Me_2_)-TB	1028	0.75	156	3.8	79.3 (3.54)	[35]
PIM-Trip(Me_2_)-TB	926	0.65	118	2.7	87.0 (3.88)	This work
PIM-EA(H_2_)-TB	845	0.62	62	2.3	71.8 (3.20)	[36,40]
PIM-Trip(H_2_)-TB	899	0.55	50	2.4	90.4 (4.03)	[29]

**Table 2:** Gas permeabilities *P_x_*, diffusivity *D_x_*, solubility coefficient *S_x_* for methanol treated films of TB-PIMs (data from aged films in parentheses and square brackets). Measurements at 25 °C and 1 bar feed pressure.

PIM	Transport Parameters	Gas Type					
		N_2_	O_2_	CO_2_	CH_4_	H_2_	He
Trip(Me_2_)-TB	*P_x_* [Barrer]	255	1002	3718	347	5446	2178
	(aged) ^a)^	(96)	(500)	(1880)	(156)	(2625)	(1134)
	*D_x_* [10^−12^ m^2^ s^−1^]	25	106	24	8	4393	7580
	(aged) ^a)^	(12.7)	(63.6)	(17.8)	(4.8)	(2487)	(4424)
	*S_x_* [cm^3^ cm^−3^ bar^−1^]	7.53	7.12	117	34.2	0.93	0.22
	(aged) ^a)^	(5.69)	(5.90)	(79)	(24.3)	(0.79)	(0.19)
EA(Me_2_)-TB [35]	*P_x_* [Barrer]	525	2150	7140	699	7760	2570
	(aged) ^b)^	(188)	(933)	(2644)	(219)	(4442)	(1630)
	*D_x_* [10^−12^ m^2^ s^−1^]	99.5	318	87	36	>7000	>10000
	(aged) ^b)^	(22.9)	(104)	(35.2)	(6.9)	(4000)	(7700)
	*S_x_* [cm^3^ cm^−3^ bar^−1^]	3.96	5.07	61.5	14.56	0.83	0.19
	(aged) ^b)^	(6.16)	(6.73)	(56.3)	23.81	(0.83)	(0.16)
EA(H_2_)-TB	*P_x_* [Barrer]	358	1673	6097	458	6088	1938
	(aged) ^c)^	(188)	(902)	(2999)	(196)	(4066)	(1367)
	*D_x_* [10^−12^ m^2^ s^−1^]	47.6	216	66.4	15.1	5635	7822
	(aged) ^c)^	(26.5)	(121)	(29.7)	(5.5)	(4074)	(6269)
	*S_x_* [cm^3^ cm^−3^ bar^−1^]	5.64	5.81	68.87	22.75	0.81	0.19
	(aged) ^c)^	(5.32)	(5.60)	(75.74)	(26.73)	(0.75)	(0.16)
Trip(H_2_)-TB [29]	*P_x_* [Barrer]	629	2718	9709	905	8039	2500
	(aged) ^d)^	(189)	(1073)	(3951)	(218)	(4740)	(1585)
	*D_x_* [10^−12^ m^2^ s^−1^]	135	462	111	48.9	7800	>10000
	(aged) ^d)^	(28.5)	(148)	(34.6)	(7.5)	(4920)	(7738)
	*S_x_* [cm^3^ cm^−3^ bar^−1^]	3.49	4.41	65.61	13.88	0.77	0.18
	(aged) ^d)^	(4.97)	(5.43)	(85.6)	(21.75)	(0.72)	(0.15)

^a)^ Age 407 days; ^b)^ Age 106 days; ^c)^ Age 119 days; ^d)^ Age 102 days.

## Supplementary Materials

The following are available online at https://www.mdpi.com/2077-0375/10/4/62/s1. Figure S1: Gel permeation chromatography trace for PIM-Trip(Me_2_)-TB.
