# Supplementary material for: Effect of Bridgehead Methyl Substituents on the Gas Permeability of Tröger’s-Base Derived Polymers of Intrinsic Microporosity
Source: Membranes (Basel). 2020 Apr 3;10(4):62. doi: 10.3390/membranes10040062 (PMC7231383; doi:10.3390/membranes10040062)
Supplement: Supplementary file 1 [file membranes-10-00062-s001.pdf]

Supporting Information for:

Effect of Bridgehead Methyl Substituents on the Gas Permeability of Tröger's-Base Derived Polymers of Intrinsic Microporosity

Richard Malpass-Evans [1], Ian Rose [1], Alessio Fuoco [2], Paola Bernardo [2], Gabriele Clarizia [2], Neil B. McKeown [1,*], Johannes C. Jansen [2,*] and Mariolino Carta [3,*]

1 EaStCHEM, School of Chemistry, University of Edinburgh, Joseph Black Building, David Brewster Road, Edinburgh, Scotland EH9 3FJ, UK; R.Malpass-Evans@ed.ac.uk (R.M.-E.); ianjamesrose@gmail.com (I.R.)

2 Institute on Membrane Technology, CNR-ITM, Via P. Bucci 17/C, 87036 Rende (CS), Italy; a.fuoco@itm.cnr.it (A.F.); p.bernardo@itm.cnr.it (P.B.); g.clarizia@itm.cnr.it (G.C.)

3 Department of Chemistry, College of Science, Swansea University, Grove Building, Singleton Park, Swansea, SA2 8PP, UK

* Correspondence: Neil.McKeown@ed.ac.uk (N.B.M.); mariolino.carta@swansea.ac.uk (M.C.); johannescarolus.jansen@cnr.it (J.C.J.); Tel. +44 131 650 4807 (N.B.M.); Tel. +44 01792 604688 (M.C.); Tel. +39 0984 49 2031 (J.C.J.)

Polymers Synthesis Procedure

9,10-dihydro-11,12-*cis*(*trans*)dichloro-9,10-ethanoanthracene[3] (1)

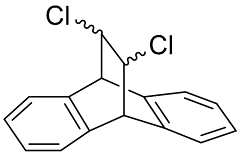

A mixture of *cis*/*trans* (1:1) 1,2-dichloroethylene (3.80 g, 39.1 mmol) and anthracene (2.00 g, 11.2 mmol) were heated to 215 ºC at 200 PSI for 5 h under microwave irradiation (300 W fixed). Excess 1,2-dichloroethylene was removed under vacuum and the black residue was dissolved in toluene (50 ml). Furan-2,5-dione (maleic anhydride) (1.00 g, 10 mmol) was added and the mixture was refluxed for 16 h to remove residual anthracene. The mixture was cooled to room temperature and the solvent was removed under vacuum. The black residue subjected to column chromatography (*n*-hexane) and the resulting yellow crystals were recrystallised from ethanol to afford the desired product 9,10-dihydro-11,12-*cis*(*trans*)dichloro-9,10-ethanoanthracene as colourless crystals(1.54 g, 50%). Mp: 190-192 ºC (Lit[3] *cis*: 203-204 ºC, *trans*: 113-114 ºC); ^{1}H NMR (500 MHz, $CDCl_3$) δ 7.44 (m, 2H), 7.37 (m, 2H), 7.30 (m, 2H), 7.23 (m, 2H), 4.57 (s, 2H), 4.52 (s, 2H); ^{13}C NMR (125 MHz, $CDCl_3$) δ 140.3, 137.9, 127.8, 127.4, 127.2, 126.8, 126.5, 125.2, 1124.6, 58.7, 52.6, 48.0, 45.4; TOF-HRMS (EI, m\z): calculated $C_{16}H_{12}Cl_2$ 274.0316 found: 274.0313 [M^+].

9,10-dihydro-9,10-ethenoanthracene (Dibenzobarrelene)[3] (2)

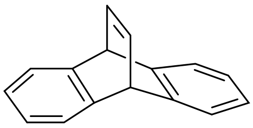

Under a nitrogen atmosphere, 9,10-dihydro-11,12-*cis*(*trans*)dichloro-9,10-ethanoanthracene (5) (6.60 g, 24 mmol) was dissolved in a refluxing mixture of anhydrous tetrahydrofuran (100 ml) and anhydrous propan-2-ol (100 ml). Sodium metal (5.49 g, 240 mmol) was added slowly in small portions and the mixture was refluxed until the sodium had been consumed. The mixture was cooled to room temperature and quenched in water. The white precipitate was filtered, washed with water and dried.

The precipitate was recrystallised from methanol to afford the desired product 9,10-dihydro-9,10-ethenoanthracene as colourless crystals(3.50 g, 71%). Mp: 125-126ºC (Lit[3] 118-119 ºC); ^{1}H NMR (500 MHz, $CDCl_3$) δ 7.39 (m, 4H), 7.11 (m, 2H), 7.06 (m, 4H), 5.25 (m, 2H); ^{13}C NMR (125 MHz, $CDCl_3$) δ 146.2, 139.5, 124.552, 123.1, 51.3; TOF-HRMS (EI, m\z): calculated $C_{16}H_{12}$ 204.0939 found: 204.0943 [M^+].

9,10-dihydro-9,10-ethanoanthracene[3] (3)

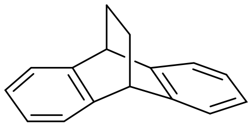

Under a nitrogen atmosphere, 9,10-dihydro-9,10-ethenoanthracene (6) (5.00 g, 24 mmol) was dissolved in tetrahydrofuran (100 ml, de-oxygenated). Raney nickel (~40 mg) and hydrazine monohydrate (24.7 ml, 24.50 g, 490 mmol) was added and the mixture was refluxed for 24 h. The colourless mixture was cooled to room temperature and filtered under nitrogen. The organic phase was extracted with chloroform and the solvent was removed under vacuum at to afford the desired product 9,10-dihydro-9,10-ethanoanthracene in a quantitative yield as colourless crystals. Mp: 143-144 ºC (Lit[3] 142-143 ºC); ^{1}H NMR (500 MHz, $CDCl_3$) δ 7.17 (m, 4H), 7.00 (m, 4H), 4.23 (s, 2H), 1.61 (m, 4H); ^{13}C NMR (125 MHz, $CDCl_3$) δ143.9, 125.6, 123.4, 44.1, 26.8; TOF-HRMS (EI, m\z): calculated $C_{16}H_{14}$ 206.1096 found: 206.1096 [M^+].

9,10-dihydro-2,6(7)-dinitro-9,10-ethanoanthracene (4)

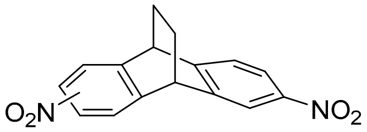

Trifluoroacetic anhydride (40.50 ml, 61.18 g, 290 mmol) was added drop-wise to a mixture of potassium nitrate ((19.61 g, 194 mmol) and 9,10-dihydro-9,10-ethanoanthracene (20 g, 97 mmol) in acetonitrile (400 ml). The mixture was stirred for 24 h, after which time a white precipitate had formed. The solvent was removed under vacuum and the residue was stirred in water then extracted with chloroform to afford a yellow solid. The crude product was subjected to column chromatography (eluent: chloroform) to afford the desired product (28.43 g, 99%) as an off-white powder. Mp: 132-133 ºC; ^{1}H NMR (500 MHz, $CDCl_3$) δ 8.18 (m, 2H), 8.08 (m, 1H), 8.06 (m, 1H), 7.49 (d, *J* = 8.10 Hz, 1H), 7.47 (d, *J* = 8.10 Hz, 1H), 4.64 (s, 2H), 1.82 (s, 4H); ^{13}C NMR (125 MHz, $CDCl_3$) δ 149.8, 149.3, 146.4, 143.9, 143.4, 124.5, 124.4, 122.1, 118.9, 118.8, 44.1, 44.0, 43.9, 25.6, 25.6, 25.5; TOF-HRMS (EI, m\z): calculated $C_{16}H_{12}N_2O_4$ 296.0797 found: 296.0800 [M^+].

9,10-dihydro-2,6(7)-diamino-9,10-ethanoanthracene (EA(H_2)-NH_2)

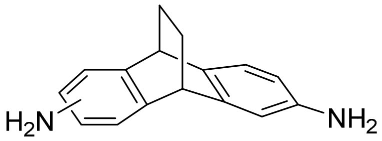

Under a nitrogen atmosphere, 9,10-dihydro-2,6(7)-dinitro-9,10-ethanoanthracene (5.00 g, 17 mmol) was suspended in diethyl ether (de-oxygenated, 200 ml). Raney nickel (~40 mg) and hydrazine monohydrate (18.90 g, 34 mmol) was added and the mixture was refluxed for 24 h. The colourless mixture was cooled in ice and filtered under nitrogen. The organic phase was extracted with diethyl ether and the solvent was removed under vacuum at 25 ºC to afford the desired product 9,10-dihydro-2,6(7)-diamino-9,10-ethanoanthracene as a colourless powder in a quantitative yield. Mp: 181-185 ºC; ^{1}H NMR (500 MHz, $CDCl_3$) δ 7.01 (d, *J* = 7.74 Hz, 1H), 7.00 (d, *J* = 7.74 Hz, 1H), 6.64 (m, 2H), 6.41 (m, 2H), 4.10 (m, 2H), 3.48 (s, br, 4H), 1.68 (s, 4H); ^{13}C NMR (125 MHz, $CDCl_3$) δ 145.8, 144.9, 144.1, 144.0, 135.3, 134.2, 123.8, 123.5, 111.8, 111.6, 111.2, 110.9, 44.4, 43.4, 42.3, 27.7, 27.2, 26.7; TOF-HRMS (EI, m\z): calculated $C_{16}H_{16}N_2$ 236.1313 found: 236.1311 [M^+].

PIM-EA(H_2) –TB was prepared according to a reported procedure [4]

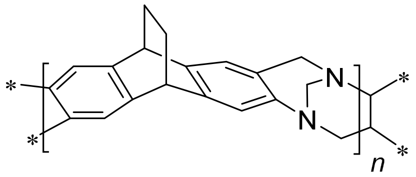

9,10-dimethylanthracene[5] (4)

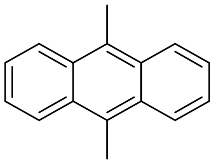

In an oven-dried nitrogen purged flask was added 9,10-dichloroanthracene (15.00 g, 60.70 mmol), PEPPSI-IPr catalyst (2.68 g, 3.95 mmol) and 1,4-dioxane (1.2 l). This was stirred under a nitrogen atmosphere for thirty minutes before methyl magnesium bromide (3 M in ether, 121.40 ml, 364.20 mmol) was slowly added. The mixture was then left stirring for 24 hours under a nitrogen atmosphere. The reaction was quenched by addition to water (2 l) and stirred for an hour before the product was extracted with ethyl acetate (3 x 250 ml). The organic solvent was removed under reduced pressure, giving the crude product as a yellow solid. This was triturated with acetone (100 ml) and the solid collected by filtration and dried under vacuum. This gave the product as a yellow powder (12.26 g, 98%). Mp 174-176 °C; Lit[5]. 181-183 °C; ^{1}H NMR (400 MHz, $CDCl_3$) δ ppm 8.35 (4H, m), 7.53 (4H, m), 3.11 (s, 6H); ^{13}C NMR (100 MHz, $CDCl_3$) δ ppm 129.9, 128.3, 125.3, 124.7, 14.1; LRMS m/z (EI, M^+) = 206.10.

Dimethyltriptycene[6] (5)

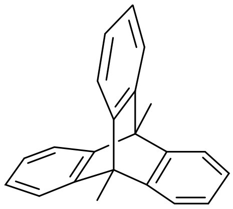

9,10-Dimethylanthracene (7.00 g, 33.98 mmol) was dissolved in dichloroethane (350 ml) at 60 °C with stirring. Benzenediazonium-2-carboxylatechloride (31.04 g, 169.90 mmol) and 1,2-epoxypropane (100 ml) were added to the solution before the mixture was refluxed at 85 °C for 40 hours under a nitrogen atmosphere. It was then allowed to cool to room temperature and concentrated under reduced pressure, giving a dark oil. The oil was added to a mixture of maleic anhydride (1.67 g, 16.99 mmol) and o-xylene (200 ml). This was heated to 110 °C for an hour before being allowed to cool to room temperature and added to water (350 ml). The crude material was extracted with dichloromethane (3 x 150 ml) and the organic solution washed with aqueous potassium hydroxide (15%, 2 x 150 ml). The organic solvent was evaporated under reduced pressure, obtaining a solid which was triturated in methanol (150 ml) giving the product as a light brown powder (7.76 g, 81%). Mp > 300 °C; Lit[6]. Mp > 300 °C; ^{1}H NMR (400 MHz, $CDCl_3$) δ ppm 7.38 (m, 6H), 7.04 (m, 6H), 2.44 (s, 6H); ^{13}C NMR (100 MHz, $CDCl_3$) δ ppm 148.5, 125.0, 120.7, 48.8, 13.8; HRMS Calc. for $[C_{22}H_{18}]^+$ m/z = 282.1408, found 282.1407.

2,6(7)-dinitro-9,10-dimethyltriptycene (6)

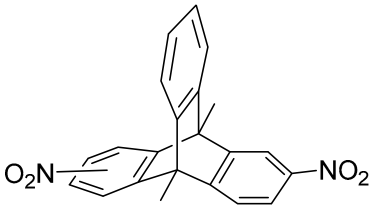

9,10-Dimethyltriptycene (2.00 g, 7.09 mmol) was dissolved in a mixture of dichloromethane (40 ml) and acetonitrile (160 ml) at room temperature. To the resulting solution was added potassium nitrate (1.43 g, 14.18 mmol) and trifluoroacetic anhydride (3.45 ml, 24.82 mmol). The reaction mixture was heated to 50 °C and stirred for 72 hours. It was then cooled to room temperature and concentrated to an orange residue under reduced pressure. The residue was added to water (300 ml) and the product extracted with chloroform (3 x 70 ml). The chloroform was removed under reduced pressure, giving the product as an off-white powder. This was passed through a silica column (4:1 dichloromethane:hexane) to give the product as a pale yellow powder (2.43 g 92%). Mp 222- 224 °C; ^{1}H NMR (400 MHz, $CDCl_3$) δ ppm 8.20 (m, 2H), , 7.99 (m, 2H), 7.51 (m, 2H), 7.44 (m, 2H), 7.15 (m, 2H), 2.54 (m, 6H); ^{13}C NMR (100 MHz, $CDCl_3$) δ ppm 154.1, 153.7, 149.3, 148.9, , 145.9, 145.5, 145.1, 126.1, 121.5, 121.4, 121.3, 116.1, 49.3, 13.5; HRMS Calc. for $[C_{22}H_{16}N_2O_4]^+$ m/z = 372.1110, found 372.1115.

2,6(7)-diamino-9,10-dimethyltriptycene (Trip(Me_2)-NH_2)

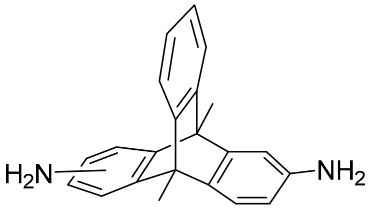

2,6(7)-Dinitro-9,10-dimethyltriptycene (3.10 g, 8.33 mmol) was dissolved in THF (100 ml) at room temperature. To this solution was added hydrazine monohydrate (6.52 ml, 83.33 mmol) and Raney nickel (catalytic amount) and the reaction mixture was heated to 60 °C and stirred for 16 hours under a nitrogen atmosphere. The mixture was then cooled to room temperature, filtered to remove the nickel and concentrated under reduced pressure to give the crude product as a yellow oil. The oil was added to water (200 ml) and the product extracted with chloroform (3 x 70 ml). The chloroform was then removed under reduced pressure, giving the product as a yellow powder (2.52 g, 97%). Mp 292-294 °C; ^{1}H NMR (400 MHz, $CDCl_3$) δ ppm 7.35 (s, 2H), , 7.09 (m, 4H), 6.74 (s, 2H) , 6.33 (s, 2H), 3.34 (br s, 4H), 2.33 (m, 6H);^{13}C NMR (100 MHz, $CDCl_3$) δ ppm 150.0, 149.6, 149.4, 148.8, 148.2, 143.5, 139.6, 138.9, 136.0, 125.6, 124.8, 124.5, 121.0, 120.8, 120.4, 120.1, 110.3, 108.9, 48.4, 47.7, 47.1,13.6; HRMS Calc. for $[C_{22}H_{20}N_2]^+$ m/z = 312.1625, found 312.1617.

PIM-Trip(Me_2) -TB

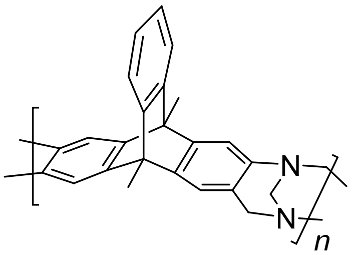

2,6(7)-Diamino-9,10-dimethyltriptycene (2.32 g, 7.44 mmol) was dissolved in trifluoroacetic acid (23 ml) with stirring at 0 °C. Once dissolved, dimethoxymethane (3.29 ml, 37.18 mmol) was slowly added over a few minutes and the reaction mixture left stirring for 96 hours under a nitrogen atmosphere. The reaction was then quenched by addition of water (100 ml) and aqueous ammonia (35%, 100 ml), before the mixture was stirred vigorously for 16 hours. The precipitated solid was then

collected by filtration, washed with water (200 ml) and acetone (200 ml) before being dried under reduced pressure. After the polymer was refluxed in acetone (100 ml) for an hour it was dissolved in chloroform (200 ml) and reprecipitated with hexane (250 ml). This was performed two additional times, before the polymer was refluxed in acetone (100 ml) and methanol (100 ml), both for 16 hours. This gave the polymer as a cream coloured powder (2.17 g, 83%). BET surface area = 926 $m^2\ g^{-1}$; total pore volume = 0.65 ml g^{-1} at P/P_0 = 0.98; TGA (nitrogen): weight loss due to thermal degradation started at 376 °C and totalled; GPC (based on polystyrene standard) M_n = 44000, M_w = 118000 $gmol^{-1}$; ^1H NMR (400 MHz, $CDCl_3$) δ ppm 6.93 (br m, 8H), 4.46 (br s, 2H), 3.92 (br s, 4H), 2.19 (br m, 6H); ^{13}C NMR (100 MHz, *solid state*) δ ppm 145.0, 124.4, 118.4, 66.9, 58.5, 47.6, 12.9.

GPC PIM-Trip(Me_2)-TB

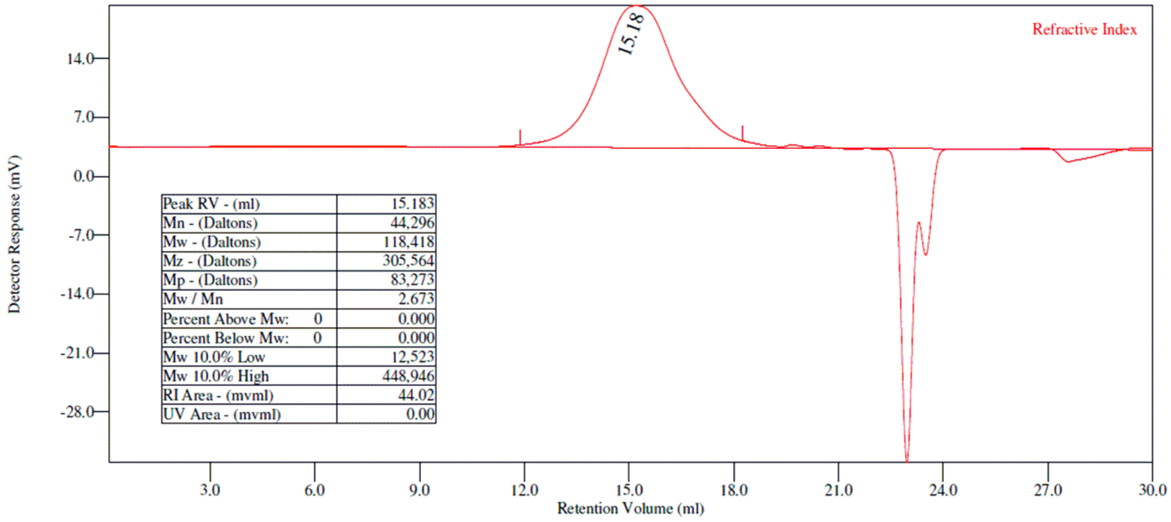

Figure S1. Gel permeation chromatography trace for PIM-Trip(Me_2)-TB

TGA Curves

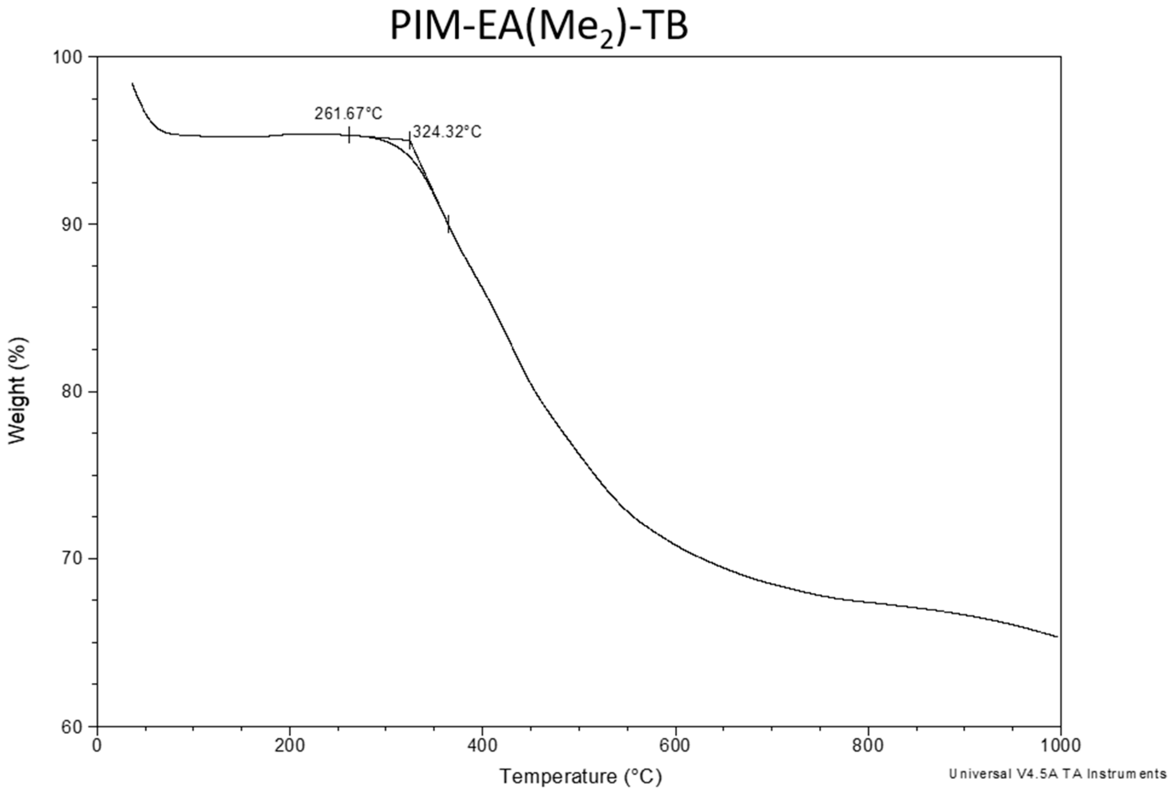
PIM-EA(Me2)-TB
100
90
80
70
60
Weight (%)
261.67°C
324.32°C
0
200
400
600
800
1000
Temperature (°C)
Universal V4.5A TA Instruments

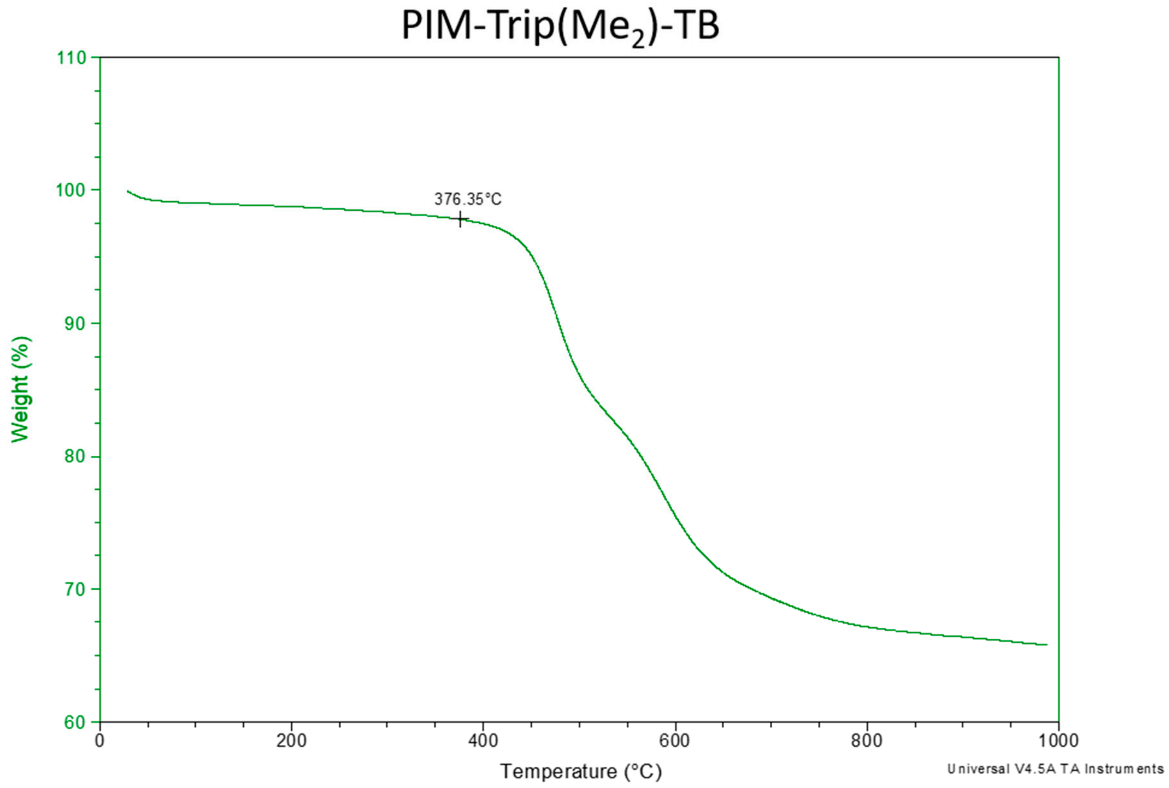
PIM-Trip(Me2)-TB
110
100
90
80
70
60
Weight (%)
376.35°C
0
200
400
600
800
1000
Temperature (°C)
Universal V4.5A TA Instruments

PIM-Trip(H_2)-TB

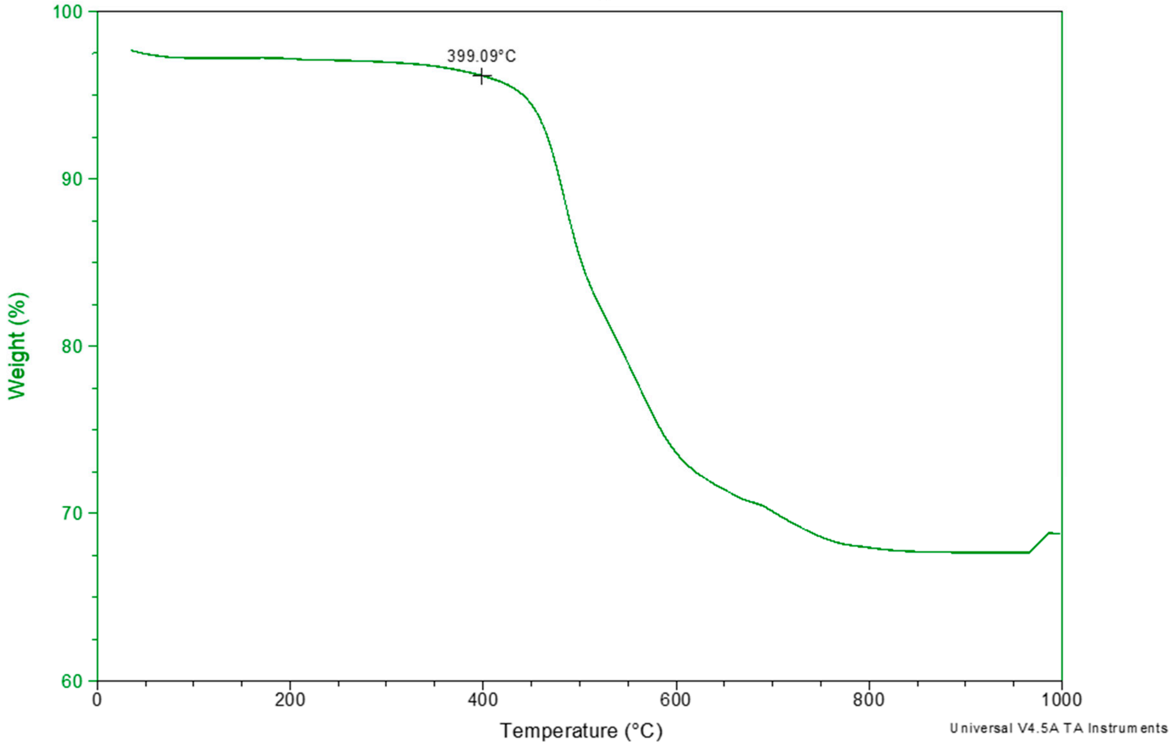

PIM-Trip(Me_2)-TB

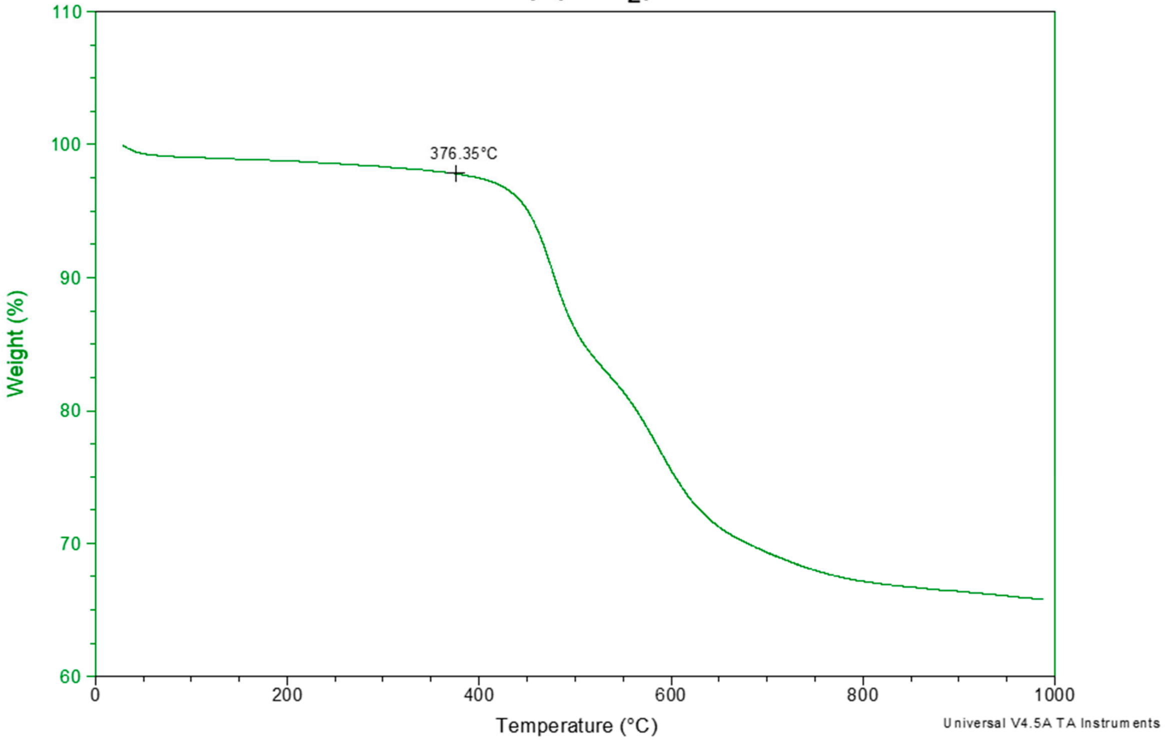

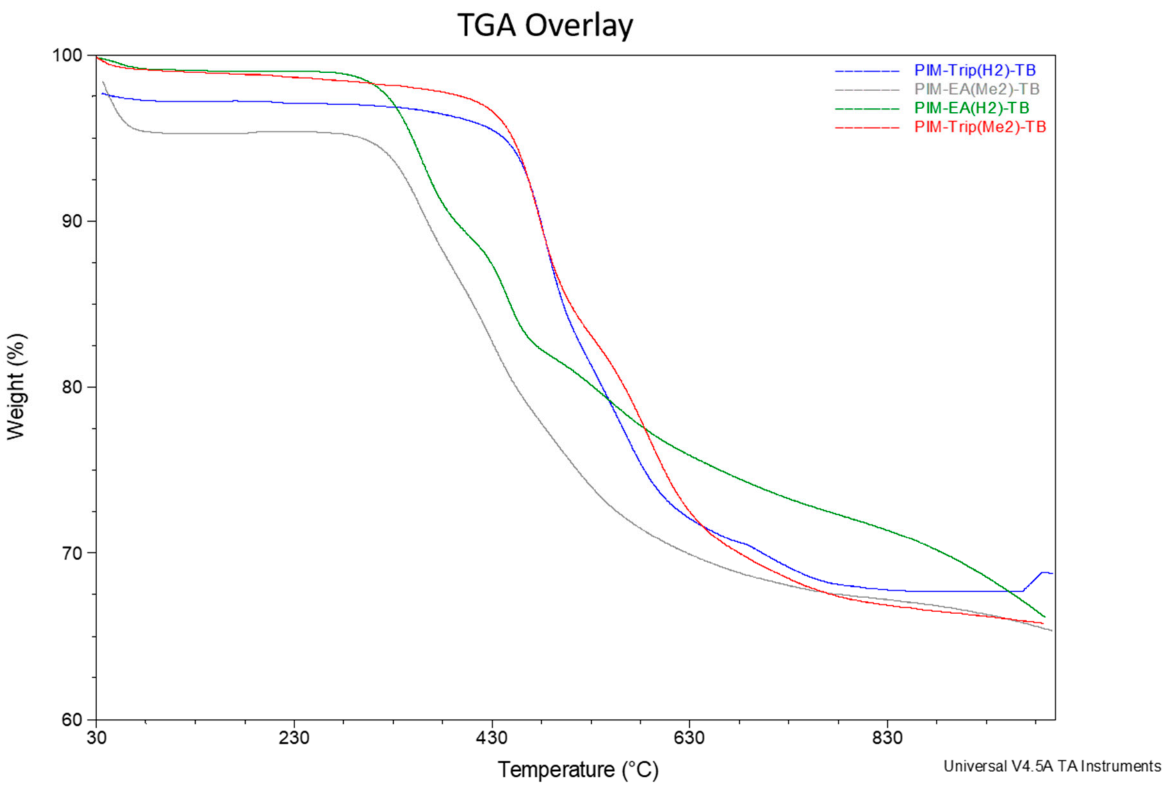

References

[1] J.C. Jansen, K. Friess, E. Drioli, Organic vapour transport in glassy perfluoropolymer membranes: A simple semi-quantitative approach to analyze clustering phenomena by time lag measurements, Journal of Membrane Science, 367 (2011) 141-151.
[2] J. Crank, The Mathematics of Diffusion. 2d Ed, 1975.
[3] S.J. Cristol, N.L. Hause, Mechanisms of elimination reactions. V. Preparation and elimination reactions of cis- and trans-11,12-dichloro-9,10-dihydro-9,10-ethanoanthracene, J. Am. Chem. Soc., 74 (1952) 2193-2197.
[4] P. Bernardo, V. Scorzafave, G. Clarizia, E. Tocci, J.C. Jansen, A. Borgogno, R. Malpass-Evans, N.B. McKeown, M. Carta, F. Tasselli, Thin film composite membranes based on a polymer of intrinsic microporosity derived from Tröger's base: A combined experimental and computational investigation of the role of residual casting solvent, Journal of Membrane Science, 569 (2019) 17-31.
[5] A. Guenzi, C.A. Johnson, F. Cozzi, K. Mislow, Dynamic gearing and residual stereoisomerism in labeled bis(9-triptycyl)methane and related molecules. Synthesis and stereochemistry of bis(2,3-dimethyl-9-triptycyl)methane, bis(2,3-dimethyl-9-triptycyl)carbinol, and bis(1,4-dimethyl-9-triptycyl)methane, J. Am. Chem. Soc., 105 (1983) 1438-1448.
[6] A. Naghipur, K. Reszka, A.M. Sapse, J.W. Lown, Formation of benzoxathiete under mild conditions and its valence tautomerism in solution to monothio-o-benzoquinone: an experimental and quantum chemical study, J. Am. Chem. Soc., 111 (1989) 258-268.
[7] V. Teplyakov, P. Meares, Correlation aspects of the selective gas permeabilities of polymeric materials and membranes, Gas Separation & Purification, 4 (1990) 66-74.
